# Endometrioma and ovarian reserve: effects of endometriomata per se and its surgical treatment on the ovarian reserve

**Published:** 2019-06

**Authors:** B Yılmaz Hanege, S Güler Çekıç, B Ata

**Affiliations:** Obstetrics and Gynecology Clinic , Koc University Hospital, İstanbul;; Department of Obstetrics and Gynecology, Koc University, İstanbul.

**Keywords:** Endometriosis, anti mullerian hormone, antral follicle count, ovarian reserve, in vitro fertilization

## Abstract

Endometrioma is the cystic lesion of ovaries originating from endometrial glands and stroma. This condition is present in 17 to 44% of endometriosis patients. The ovarian reserve is decreased in women with endometriomas, as compared to similarly aged healthy women or women with other benign ovarian cysts. Some data suggest women with endometrioma experience a faster decline in AMH than age matched healthy women. Multiple well-designed studies consistently demonstrate that surgical excision of endometrioma is associated with a decline in the ovarian reserve. Recent studies with long term follow up suggest some recovery in the markers of ovarian reserve, but they almost never reach preoperative levels. The energy modality and choice of hemostatic method may be important. Limited data suggest ablation of the cyst wall with plasma energy is associated with less harm to reserve with similar recurrence rates as compared with excision and bipolar coagulation. In conclusion, bipolar diathermy seems to be the most harmful hemostatic method to ovarian reserve and its use should be cautiously minimized.

## Introduction

Endometriosis is characterized by the presence of endometrial tissue outside the uterine cavity and is estimated to affect around 2-10% of women in their reproductive years (Nnoaham et al., 2011). Ovarian endometriomas are present in 17% to 44% of women with endometriosis (Redwine, 1999; Vercellini et al., 2003). Even though a causal relationship between endometriosis and infertility has not been proven, subfertility is the presenting symptom for 30-50% endometriosis patients. The disease can affect fertility by altering tuba-ovarian function, gamete transport, endometrial receptivity and inducing an inflammatory reaction within the peritoneal cavity that can lead to alteration in sperm quality and function (Yoshida et al., 2004; Gupta et al., 2006; Mansour et al., 2009, Matsuzaki and Schubert, 2010; Young et al., 2013). While the presence of an endometrioma does not seem to affect ovulation per se, there are concerns about both a qualitative effect on oocytes and a quantitative decline in ovarian reserve (Leone Roberti Maggiore et al., 2015). This narrative review aims to examine the effect of endometriomas per se and their surgical treatment on ovarian reserve.

## Endometriosis and Ovarian Reserve

### 

The ovarian reserve reflects the reproductive potential and oocyte function, both qualitatively and quantitatively in a patient (Practice Committee of the American Society for Reproductive Medicine, 2015a,b). Endometriomas can affect ovarian reserve in two ways: (i) impairing circulation in the ovarian cortex by the compression of the cyst and thus causing follicle loss; and/or (ii) the inflammatory setting within the cyst walls leading to follicular damage (RCOG, 2018).

The most reliable and widely used quantitative ovarian reserve markers are antral follicle count (AFC) and serum anti-Mullerian hormone (AMH) levels. Number of oocytes retrieved (NOR) during assisted reproductive technology (ART) cycles can also be regarded as a marker for ovarian reserve.

### Antral Follicle Count

AFC is defined as the total number of antral follicles between 2-10 mm observed in early follicular phase by transvaginal sonography (Sanchez et al., 2014). AFC is a commonly used and reliable sonographic ovarian reserve marker. The main limitation of AFC is its intercycle variation of individual ovaries and the challenge of obtaining a high resolution image of ovaries in the presence of an endometrioma. Endometriotic cells in the cyst produce high concentrations of iron which leads to reactive oxygen species (ROS) (Sanchez et al., 2014). ROS and transforming growth factor beta induce fibrosis, which distorts pelvic anatomy. Fibrosis and anatomic distortion may negatively affect the quality of the images produced, by increasing the distance between the ultrasound probe and the ovary. As a result, the AFC may be an underestimation of the real value in the presence of endometriomas.

Lima et al. (2015) retrospectively reviewed the records of 37 women with a unilateral endometrioma who undervent an ART cycle. They suggested that AFC in ovaries with endometrioma is likely to be underestimated, since they could retrieve a higher number of oocytes than expected based on the AFC. Despite the fact that AFC was lower in ovaries with endometriomas compared to healthy ovaries median AFC: 3.0 (25 th - 75 th percentile; 1.0-6.0) vs 5.0 (25 th -75 th percentile, 2.0-6.5), p=0.001, respectively. The total number of oocytes collected was similar (median:2.0, (25 th -75 th percentile: 0.5 - 5.0) to 2.0 (25 th - 75 th percentile, 0.0-4.0), p=0.60. The underestimation of AFC is attributed to the impairment of the transvaginal probe resolution due to longer path length (the capsula of the cyst wall) and higher-frequency waves resulting in greater attenuation and making it difficult to detect smaller follicles. These findings comprise evidence for AFC being underestimated in the presence of an endometrioma as it reduces visualization of small follicles at the beginning of the cycle.

A meta-analysis of two retrospective studies, reported that women with in situ endometrioma had similar AFC compared to women without endometriosis (standardized mean difference [SMD]: - 0.02; confidence interval (CI): - 0.21 to 0.18) (Bongioanni et al., 2011; Benaglia et al., 2013; Hamdan et al., 2015). In contrast, a prospective study involving 60 women with an average age of 30 years, reported that women with endometrioma had significantly lower AFC compared to healthy women with no endometriomas (9.73 ± 4.77 vs. 14.7 ± 4.1, respectively, p < 0.01) (Uncu et al., 2013).

At present, AFC by ultrasound is the only way of assessing reserve of each ovary for most cases. However, based on the current recommendations, AFC has limited value in women with ovarian endometriomas or previous ovarian surgery (Broekmans et al., 2010).

### Serum Anti Mullerian Hormone Level

Serum AMH is a reliable marker of ovarian reserve, without the limitations of ultrasound and less intercycle variations than other hormonal serum parameters, such as follicle stimulating hormone (FSH) levels. In a recent systematic review and meta-analysis, a total of 17 studies including a total of 968 women with endometrioma and 1874 controls were pooled together. AMH levels were significantly lower in the endometrioma group compared to women with non-endometriotic benign ovarian cysts and/or healthy ovaries (mean difference: -0.84 ng/ml; CI: -1.16 to -0.52; P<0.01) (Muzii et al., 2018). In the secondary analyses, women with endometriomas were separately compared with women with non-endometriotic ovarian cysts (mean difference: -0.61 ng/ml; CI: -1.37 to -0.32) and women with normal ovaries (mean difference: - 0.61 ng/ml; CI -0.99 to -0.24). Both comparisons showed significantly lower AMH in endometrioma patients. These findings strongly suggest that the mere presence of endometrioma per se is associated with decreased AMH levels in women with endometrioma.

Kasapoglu et al. (2018) compared the longitudinal decline of AMH levels over six months, between 40 women with endometriomas and similarly aged 40 healthy women. None of the women were on any medication that could affect AMH levels. AMH levels were found to decline faster in the endometrioma group (median decline: -26.4% (25 th and 75 th percentile -11.36% to -55.41%) vs. -7.4% (25 th and 75 th percentile: 11.98%, to -29.33%), in the endometrioma and control groups, respectively). The increased rate of decline can be an explanation for the lower AMH levels observed in women with endometrioma in previous cross sectional studies (Uncu et al., 2013).

### Number of Oocytes Collected from Endometrioma Containing Ovaries

A meta-analysis of studies assessing effect of endometrioma on ART outcome, reported a lower mean NOR from women with endometrioma than from women without endometrioma (SMD: -0.23; CI [-0.37, -0.10]) (Hamdan et al., 2015). However, endometrioma increases the risk of failure to aspirate all available follicles during oocyte retrieval by 3.6 fold (Dan and Limin, 2013). Therefore, it is unclear whether the small but statistically significant decrease in NOC is due to a decline in the number of growing follicles and oocytes or the relative difficulty of oocyte pick-up procedures.

In conclusion, women with endometrioma have an increased rate of AMH decline and lower serum AMH levels compared to similarly aged healthy women. Women with endometrioma also have a lower NOC during ART cycles. Although the observations on AFC are conflicting, the value of AFC as a marker of ovarian reserve is limited in the presence of an endometrioma. In our opinion, the overall available evidence suggests that endometrioma per se is associated with a decrease in ovarian reserve (Table I).

**Table I:** — Summary of effect of endometrioma and its surgical treatment on ovarian reserve.

	Antral Follicle Count	Anti-Mullerian Hormone	Number of oocytes collected	Comment
Endometrioma	Has limited value in the presence of endometrioma, due to limited visibility of antral follicles.	Studies consistently show decreased AMH levels.	Less oocytes are collected from endometrioma containing ovaries.	The presence of endometrioma is associated with a decline in ovarian reserve.
Surgical treatment	A decline in AFC following surgery is not unequivocally demonstrated.	Studies consistently show a permanent decline in serum AMH.	Studies consistently show that less ocoytes are collected from operated ovaries.	Surgical treatment of endometrioma is associated with a decline in ovarian reserve. However, different techniques can have different effects.

### Antral Follicle Count

Muzii et al. (2014) in a meta-analysis of 9 studies and 511 patients, reported that AFC remains unchanged after endometrioma excision (median difference: 0.10; CI: -1.45, 1.65). However, a subanalysis of 145 patients revealed that AFC in the operated ovary is indeed lower than the contralateral ovary (median difference: -1.40; CI: -2.27, -0.52, p=0.002). Yet, the authors commented that cystectomy does not affect ovarian reserve. Nevertheless, as mentioned before, AFC is not a reliable marker in the presence of an endometrioma (Ata and Urman, 2014). As mentioned in the study by Lima et al. (2015a,b) preoperative AFC might be underestimated than it really is in the presence of an endometrioma. Following endometrioma excision and restoration of pelvic anatomy, sonographic quality is possibly improved enabling a more accurate AFC. Therefore, eventhough the AFC is decreased in reality, due to the improvement in ultrasound resolution after surgery, the impact may be underestimated. We strongly believe that AFC is not a reliable marker to assess the change in ovarian reserve in the context.

### Serum Anti Mullerian Hormone Levels

Raffi et al. (2012) in a metaanalysis of eight studies and 237 patients, reported a statistically significant decline in AMH levels following endometrioma surgery (1.13 ng/ml, CI; 0.37 - 1.88). The decline in AMH levels was significant even after unilateral cystectomy (30% decline, SMD: -0,96 ng/mL; CI: -0,22, -1,70). The decline is reported to be 44% after bilateral endometrioma excision. Somigliana et al. (2012) published a similar systematic review with 11 studies in the same year, but did not pool the data due to the heterogeneity of original studies. Nine of the eleven studies reported a decrease in AMH levels after surgery, while only two studies reported otherwise. The authors concluded that endometrioma excision leads to a decline in AMH levels.

Results of most recent studies regarding postoperative AMH changes are consistent with these two systematic reviews and suggest that endometrioma excision leads to significant decrease in AMH levels (Raffi et al., 2012, Somigliana et al., 2012). Goodman et al. (2016) prospectively compared 58 patients with endometrioma with 29 women without endometriosis; preoperatively, AMH levels were significantly lower in the presence of an endometrioma (1.8 ng/mL; CI, 1.2 - 2.4, vs. 3.2 ng/mL; CI: 2.0 – 4.4). One month after endometrioma excision AMH levels were significantly lower than preoperative levels (decline by -48%; CI; -54%, -18%; P<0.01, mean AMH decreased from 1.77 ng/ml to 1.12 ng/ml). At the sixth month after surgery, despite some increase over the values at the first month, AMH levels were still lower than the preoperative levels, albeit short of statistical significance (1.41 ng/ml, CI: 0.97 -1.85, compared to preoperative levels p=0.22).

In a prospective study of 25 patients undergoing unilateral endometrioma excision, serum AMH levels decreased by 24% in the first month and persisted at this level on the postoperative sixth month (Urman et al., 2013). Another prospective study including 30 women with a long term follow up reported a significant gradual decline in AMH levels in the first and sixth months (control and endometrioma excision patients respectively; preoperative AMH: 2.81 and 2.15 ng/ml, postoperative 1 month AMH: 2.07 and 1.47 ng/dl, postoperative 6 month: 1.82 and 1.29 ng /ml) (Uncu et al., 2013). Both studies suggest that the AMH decline is permanent.

Alborzi et al. (2014) followed up 121 women with unilateral and 72 women with bilateral endometrioma excision up to 9 months, and reported that both groups had a significant AMH decline in the postoperative 1st week. Even though AMH levels recovered to some extent by the postoperative third month, they were still significantly lower than the preoperative levels by the ninth month. AMH decline was more prominent following bilateral endometrioma excision compared to unilateral excision (preoperative AMH 4.19 ± 3.71 vs 3.29 ± 3.28 ng/ml; 1. week AMH 1.99 ± 2.08 vs 1.03 ± 1.40 ng/ml; p<0.05, 9.month 2.18 ± 1.87 vs 1.19 ± 1.43 ng/ml). Other studies revealed a similar decline in AMH levels one year after surgery, particularly following bilateral endometrioma excision (Shao et al., 2016; Kovacevic et al., 2018). However, these findings cannot be regarded as a solid proof for a significantly higher decline following bilateral endometrioma excision, because the change in AMH is assessed in absolute values, and the higher preoperative AMH levels in women with unilateral endometriomas could have reflected on the following months after surgery.

The overall available evidence suggests that endometrioma excision leads to a significant and permanent decline in serum AMH levels.

### Number of Oocytes Collected in Ovaries with Endometrioma

The above mentioned meta-analysis on the effect of endometrioma on ART outcome, reported that mean NOC was significantly decreased in the surgically treated ovary compared to the contralateral normal ovary (mean difference: -2.59; CI: -4.13, -1.05), 4 studies, 222 cycles) (Hamdan et al., 2015). Based on nine studies with 810 cycles, the NOC from ovaries which underwent endometrioma excision were similar with NOC from ovaries with in situ endometriomas (SMD: -0.17, CI: -0.38 to 0.05) (Hamdan et al., 2015).

In our opinion, this can be explained by the following: i) the challenge of demonstrating a further decrease in ovarian reserve as statistically significant while it is already decreased due to the presence of an endometrioma per se, and/or ii) surgery rendering oocyte retrieval easier and thus leading to aspiration of more follicles during the procedure, despite the presence of less follicles following surgery than before surgery. A more recent meta-analysis with a larger sample size, i.e. data from 16 studies and 2299 patients, lends credit to this as it revealed that NOC from operated ovaries were significantly less than NOC from endometrioma containing ovaries (median difference: -1.78; % 95 CI: -2.38 ila -1.17, p<0.0001) (Tao et al., 2017). Similar results were obtained by sensitivity analysis where heterogenity is reduced (Tao et al., 2017). Yet, both meta-analyses reported similar live birth rates for women with in situ endometriomas and women who underwent endometrioma excision, despite decreased oocyte numbers in the latter (Hamdan et al., 2015, Tao et al., 2017).

## Determinants of surgery related decline in ovarian reserve

Age at the time of surgery, cyst size and inadvertent removal of healthy ovarian tissue during surgery have been investigated as determinants of surgery-related decline in ovarian reserve. Overall, age and cyst size do not seem to be related with the rate of AMH decline (Raffi et al., 2012, Somigliana et al., 2012).

The effect of laterality is controversial since there are contradictory results (Hirokawa et al., 2011; Celik et al., 2012; Uncu et al., 2013; Alborzi et al., 2014). In the two studies, in which the decline in AMH levels following bilateral endometrioma excision did not reach statistical significance, the absolute decline was greater in women who underwent bilateral endometrioma excision. This could be due to small number of cases in the study groups (Celik et al., 2012; Uncu et al., 2013; Salihoglu et al., 2016). In our opinion, everything else being equal, bilateral endometrioma excision inevitably affects ovarian reserve more than unilateral cyst excision.

In both Çelik et al. (2012) and Uncu et al. (2013) studies, there was a positive correlation between preoperative AMH levels and postoperative AMH decline. Women with higher preoperative AMH levels, the absolute decline in AMH levels was greater than women with lower preoperative AMH levels. Possibly an ovary with a higher reserve has a higher primordial follicle intensity and thus, during the intervention, inadvertent removal of cortex and/or surgical damage lead to a higher number of follicles being lost, resulting in a bigger decline in AMH production (Celik et al., 2012, Uncu et al., 2013). However, despite a greater loss, women with a higher reserve tend to have an acceptable postoperative serum AMH levels.

One of the factors that damage ovarian reserve is the method used for hemostasis after cyst excision. Sutures, hemostatic gel or bipolar cauterization (BC) are commonly used for hemostasis. The disadvantages of suturing are the need for expertise and the potential ischemia due to an increase in intraovarian pressure after suture tightening. Hemostatic gels are expensive and can lead to serious adverse effects like bowel obstruction or thromboembolism (Misirlioglu et al., 2018). BC is inexpensive, easy and effective but should be used carefully to limit thermal injury.

A meta-analysis pooled six studies that compared the effects of BC, hemostatic gel and sutures on ovarian reserve after endometrioma excision. BC seems to be the most destructive hemostatic modality on ovarian reserve (Ata et al., 2015). Other studies published subsequently support this finding. In a recent randomized controlled study involving 109 patients, Asgari et al. (2016) compared BC to suturing after unilateral endometrioma excision and reported that AMH is decreased in both groups 3 months after surgery but the decrease was higher in BC group. Likewise, Choi et al. (2018) prospectively compared hemostatic gel and BC following endometrioma excision, and reported a significantly higher rate of AMH decline with BC.

Plasma energy is another modality used for ablation of endometriomas. Some data suggest limited thermal damage to surrounding ovarian tissue following ablation with plasma energy (Auber et al., 2011, Roman et al., 2011). Roman et al. (2011) retrospectively compared AFC between 30 women with unilateral endometriomas larger than 30 mm in diameter. None of the women had a history of ovarian surgery and they were all given suppressive hormonal therapy until desire for pregnancy. Fifteen women underwent cyst excison followed by BC, while the other 15 underwent ablation of endometrioma with plasma energy. Women in both groups were similar for age, cyst diameter and side, as well as the revised American Society of Reproductive Medicine (rASRM) scores. Despite similar AFC in the endometrioma containing ovary before surgery (6.8 ± 3.5 vs 8 ± 5.3, in plasma energy and BC groups, p = 0.47, respectively), women in the plasma energy ablation group had significantly higher postoperative AFC in the operated ovary than women in the cystectomy and BC group, 5.5 ± 3.9 vs 2.9 ± 2.4, p=0.03, respectively). These encouraging results need to be replicated in randomized controlled trials.

Vaporization of cyst wall by CO_2_ laser is compared with laparoscopic cyst excision in a randomized controlled trial including 30 reproductive age women in each group (Candiani et al., 2018). Three months after surgery, women in both groups had an increased AFC as compared to baseline. While the increase in AFC was significant in CO_2_ laser vaporization group (from 3.6 ± 1.9 at baseline to 8.6 ± 4.2, p<0.01), the difference was short of statistical significance in the excision group (from 4.1 ± 2.2 at baseline to 6.3 ± 3.5, p = 0.06). While serum AMH level was significantly decreased in the excision group (from 2.6 ± 1.4 ng/mL at baseline to 1.8 ± 0.8 ng/mL, p=0.01), it remained unchanged in the CO2 vaporisation group (from 2.3 ± 1.1 ng/mL at baseline to 1.9 ± 0.9 ng/mL, p=0.09). Inter-group comparisons showed significantly different ΔAFC and ΔAMH values between the groups, both in favor of CO_2_ laser vaporisation. Experience with CO_2_ laser is limited and more studies are needed to confirm these findings.

The Working Group of European Society for Gynecological Endoscopy, European Society of Human Reproduction and Embryology and World Endometriosis Society has published recommendations on the technical aspects of different surgical methods for endometriomas in reproductive age women (Saridogan et al., 2017). These recommendations suggested approaches to minimise the detrimental impact of surgery for endometriomas.

## Effect of Advanced Endometriosis Surgery on Ovarian Reserve

There is less information on the effect of extraovarian endometriosis on ovarian reserve. Hirokawa et al. (2011) reported that there is a significant relationship between the revised American Society for Reproductive Medicine (rASRM) scores and decrease in serum AMH levels. There is a significantly higher decrease in AMH after surgery for advanced endometriosis compared to early stage disease. A possible explanation is deterioration in ovarian blood flow during extensive adhesiolysis and dissection. However, it is very difficult, if not impossible, to reliably pool data from women undergoing endometriosis surgery which may include salpingectomy and separation of adherent ovaries, as these procedures may influence the degree of ovarian damage.

## Fertility Preservation in Endometriosis Patients

Endometriosis is a chronic condition with a high rate of reccurence in the absence of suppressive treatment. Patients tend to redevelop endometriomas and/or to be reoperated several times. The disease is not necessarily progressive, but it is not yet possible to foresee in whom and when the disease will be progressive. Coccia et al. (2011) reported that patients operated for bilateral endometriomas tend to become menopausal earlier than others. Similarly, a study on women with poor ovarian reserve (POR), reported that women with previous endometriosis surgery and POR are significantly younger than women with POR patients but no history of surgery (Hong et al., 2017).

The mere presence of endometriomas and their surgical treatment pose a threat to ovarian reserve. In our opinion, it would be prudent to carefully inform patients about the risks of premature ovarian failure. But care should be taken not to cause unnecessary anxiety, since many patients will not experience this. Counselling should be individualized based on age, number of children, plans for future pregnancy, possible need for surgical treatment and current ovarian reserve status. They should be appropriately informed about fertility preservation methods.

## Conclusions

Women with endometrioma have significantly lower serum AMH levels and seem to experience a more rapid decline in serum AMH levels than age matched counterparts, suggesting an harmful effect of endometrioma per se on ovarian reserve. It is more difficult to document the same with AFC, which has limitations as a marker of ovarian reserve in the presence of endometrioma and endometriosis. Available evidence consistently suggests that surgical treatment of endometrioma inflicts harm on ovarian reserve as well. However, different surgical techniques involving plasma energy or CO_2_ laser yielded promising preliminary results, which need to be confirmed in future studies.
